# Dynamics of Membrane Trafficking Downstream of B and T Cell Receptor Engagement: Impact on Immune Synapses

**DOI:** 10.1111/j.1600-0854.2009.00913.x

**Published:** 2009-04-22

**Authors:** Maria-Isabel Yuseff, Danielle Lankar, Ana-Maria Lennon-Duménil

**Affiliations:** INSERM U932, Institut Curie12 rue Lhomond, 75005, Paris, France

**Keywords:** immune synapse, polarity, B cell receptor, T cell receptor, cytoskeleton, membrane trafficking

## Abstract

The onset of an adaptive immune response requires the activation of T and B lymphocytes by antigen-presenting cells, through a specialized form of intercellular communication, known as the immunological synapse (IS). In B lymphocytes the IS promotes efficient recognition and acquisition of membrane-bound Ags, while in T cells, it modulates the T cell response upon exposure to peptide-major histocompatibility complexes. In this review, we highlight the similarities that determine B and T cell activation, focusing on immune receptor downstream signaling events that lead to synapse formation. We stress the notion that polarization of T and B lymphocytes characterized by global changes in cytoskeleton and membrane trafficking modulates synapse structure and function, thus determining lymphocyte effector functions and fate.

## Adaptive Immunity

The entrance of pathogens into multicellular organisms triggers an immunological response involving the coordinated action of effector cells and molecules destined to destroy and eradicate them. Although the initial immune response, termed innate, is rapid and relatively non-specific, the following phase is highly specific and is referred to as adaptive immunity. The onset of the adaptive immune response begins when pathogen-derived antigens are transported by dendritic cells (DCs) from infected peripheral tissues to lymph nodes for presentation to T cells, subsequently leading to activation and clonal expansion of T help cells (CD4+) and cytotoxic T cells (CD8+). The latter T cell subset, once activated, will migrate to the sites of infection to destroy Ag-bearing cells, accompanied by activated CD4+ T cells, which provide helper functions to CD8+ cells and recruit the effectors of inflammation. In addition, activated CD4+ T cells migrate toward lymph node follicles to interact with B cells that have specifically internalized and processed the Ag. This cellular communication known as T–B cooperation (1) is required for B cells to develop into germinal centers, form high-affinity antibody-producing plasma cells and to further develop B cell memory (2). Thus, a proper immune response requires the concerted activation of B and T cells, which must be tightly regulated to ensure efficacy, specificity and memory. In this review, we address the principal signaling and membrane-trafficking events triggered by immune receptor engagement that govern B and T cell activation, through specialized interactions, known as immunological synapses (ISs).

## T and B Cells Form Synapses

### Cellular interactions involved in the IS

The activation of T and B cells requires their appropriate interaction with antigen-presenting cells (APCs), by engagement of their immune receptors with the corresponding antigens. This close communication takes place through specialized signaling areas, which are referred to as the IS (3). Although it was initially observed in activated CD4 T cells, ISs also occur in naïve CD4 cells (4), and CD8 T cells, both during initial priming by APCs (5) and during recognition and killing of target cells by cytotoxic CD8 T cells (6). To constitute an IS the T cell receptor (TCR) must recognize Ag in the form of processed peptides loaded onto major histocompatibility complex (MHC) molecules, which may be present on the cell surface of professional APCs or of infected cells. The interaction of the TCR with MHC class I–peptide complexes triggers the activation of cytotoxic CD8+ T cells, whereas MHC class II–peptide complexes, in addition to costimulatory signals, lead to CD4+ T helper cell activation. In B cells, the interaction of the B cell receptor (BCR) with Ags tethered at the surface of APCs leads to the formation of a synapse, which does not only control their activation but also the subsequent gathering and acquisition of the membrane-bound Ag for processing and presentation onto MHC class II molecules to CD4+ T cells (7). The recruitment of receptors and signaling molecules to the IS directly impacts the effector functions of lymphocytes and therefore, must be tightly regulated.

### Fundamental features of B and T cell ISs

The IS forms on the contact area of B and T cells interacting with APCs. In spite of the differences in the nature of the Ags recognized by each type of receptor, T and B cells form similar IS that display common structural and functional properties. In both cases a highly dynamic structure is formed, integrating two concentric regions referred to as the central supramolecular activation cluster (cSMAC), where TCR or BCRs, respectively are enriched, and the peripheral SMAC (pSMAC) that contains adhesion molecules such as LFA-1 bounds to its ligand ICAM-1 (7,8). Engagement of integrins with their respective ligands, on the surface of APCs, is required for the early stages of Ag recognition and facilitate both T and B cell activation by promoting adhesion to the target cell (9,10).

A mature IS comprises the reorganization of cell surface receptors and the recruitment of cytoplasmic signaling effectors, which are temporally and spatially organized to control cytoskeletal rearrangements and membrane-trafficking events. In turn, as discussed below, changes in the cytoskeleton interface will modify composition, function and fate of the IS, ultimately determining lymphocyte activation.

## Receptor Trafficking and Synapse Formation

### Cell receptors and signaling

**Figure 1:**
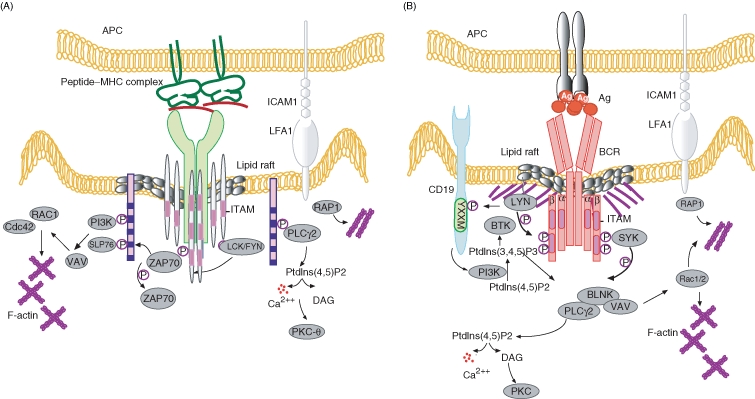
Early stages of B and T cell activation: formation of signaling platforms Engagement of B and T cell immune receptors triggers the activation of Src-family tyrosine kinases that lead to the phosphorylation of ITAMs within the cytoplasmic domains of immune receptor subunits and to the further recruitment of adaptor and signaling molecules, forming signaling platforms that involve lipid microdomains. A) T cell activation, initiated by TCR engagement with MHC–peptide complexes, activates Lck and Fyn, which results in phosphorylation of CD3 ITAM modules, leading to the sequential activation and recruitment of signaling proteins, such as ZAP70, LAT and SLP-76. B) In B cells, BCR recognition of Ag, most likely in a membrane-bound form, leads to the activation of Lyn, which phosphorylates the ITAM motifs on the Igα/Ig*β* BCR subunits, an event that further determines the recruitment and phosphorylation of Syk, Btk and adaptors, such as BLNK and CD19. In both cases downstream effectors of receptor signaling lead to intracellular calcium release and production of secondary messengers, such as diacylglycerol (DAG) and inositol-1,4,5-trisphosphate (InsP3), which activate PKC -θ in T cells and PKC in B cells, determining effector functions. The main pathway of TCR-dependent actin remodeling involves signaling through LAT and SLP76, AKT and Vav-1, which are activated and stabilized at the IS. Vav-1, a GEF for Rho-GTPases triggers the activation of Cdc42 and Rac1, which promote actin polymerization by Wasp and WAVE2, respectively. The BCR-mediated spreading and contraction of B cells depend on actin cytoskeleton, which also rely on activation of Rac1 and 2. Additionally, in B and T cells, Rap1 activation through LFA-1 engagement promotes actin polymerization, most likely in a cooperative manner with Rho-GTPases.

In addition to their structural similarities, TCR and BCR display common functional properties. Both receptors contain a central Ag recognition subunit, belonging to the superfamiliy of immunoglobulins, which are associated with a signal-transducing complex. The downstream signaling cascades resemble each other in terms of signaling molecules involved and their mode of activation (Figure 1).

The TCR multisubunit complex comprises an α/*β* heterodimer, which is involved in antigen recognition, and a signaling module formed by three pairs of CD3 invariant chains: the heterodimers *γδ* and *γε* and the homodimer ζ-*ζ*(11). The early events of T cell activation include phosphorylation of tyrosine residues of the immunoreceptor tyrosine-activation motifs (ITAMs) within the cytoplasmic tails of CD3 by Lck, an Src family kinase (12). This triggers calcium signaling and the recruitment of tyrosine kinases, such as ZAP-70, which are docked on the phosphorylated ITAMs and in turn activate important signaling molecules, such as protein kinase C-*θ* (PKCθ), thereby inducing cytokine production and controlling the activation of genes involved in T cell survival and effector functions (13) (Figure 1).

The Ag recognition subunit of the BCR corresponds to a plasma membrane immunoglobulin (Ig), which is coupled to a signaling module formed by the Igα/Igβ dimer (14). In contrast to the TCR, Ags recognized by the BCR do not require coupling to MHC molecules and are diverse in nature. Under this context, B cells respond to soluble Ags and ones encountered in a membrane-bound form (7) interactions that most likely determine the outcome of B cell activation. The signal transduction cascades through the BCR trigger phosphorylation of tyrosine residues within the ITAMs of the Igα/Ig*β* cytosolic domains, by Src-family kinases, such as Lyn (15), which, in turn, lead to the activation of Syk (16) and calcium signaling (Figure 1). This promotes the recruitment and activation of signaling molecules that control gene transcription, as well as reorganization of the cytoskeleton and BCR trafficking, ultimately leading to the formation of a specialized compartment devoted to Ag processing (see below).

How immune receptor engagement drives IS formation during T and B cell activation remains incompletely understood. Most studies have addressed the functional role of the IS on immune receptor signaling in T cells, leading to some controversy. The cSMAC was proposed to play a role in enhancing TCR signaling, based on the observations that signaling molecules, such as PKC*θ* and src-kinases, are enriched in the cSMAC and inhibitory molecules excluded (8). However, studies showing that TCR signaling occurs before cSMAC formation (4) and is rather initiated in peripheral microclusters (17) have challenged this model.

The role of the IS in B cell signaling has been less explored. Similarly, to their T cell counterpart, early signaling events in B cells precede the formation of a mature IS, as observed by total internal reflection microscopy (TIRFM) during B cell activation with Ag on a lipid bilayer (18). These studies revealed that upon initial contact with Ag, BCR-enriched microclusters are formed, which are active in signaling, as detected by calcium response and recruitment of signaling molecules, such as Syk. Interestingly, these signaling-competent microclusters resemble those detected in early phases of TCR stimulation (19), illustrating that, in addition to common structural features, BCR and TCR use similar mechanisms to initiate signaling cascades leading to IS formation.

How receptor signaling is translated to coordinate membrane trafficking for mature synapse formation remains largely unknown. Recruitment of lipid rafts and cytoskeleton polarization toward the IS are the key events that not only contribute to synapse formation, but also modulate downstream membrane trafficking, which in turn, will determine IS composition and function.

### Role of lipid rafts in receptor signaling

The lipid microenvironment plays a key role in the temporal and spatial coordination of the assembly of both TCR- and BCR-signaling modules. Stimulation of the immune receptors quickly triggers their incorporation into low-density detergent insoluble membrane domains, termed lipid rafts (20,21). This is accompanied by the recruitment of kinase-signaling molecules and lipid-modifying enzymes, such as PI3K and, PLC*γ* thus highlighting their role as platforms in initiating signaling cascades of T and B cells. Accordingly, disruption of lipid rafts significantly impairs the early steps of T cell activation (22), therefore, raft integrity is clearly a prerequisite for efficient TCR signal transduction. During T cell activation, small pre-assembled lipid rafts were shown to traffic to the IS, partly through actin- and myosin-dependent processes (23), thus providing an efficient way to target the signaling molecules necessary for synapse formation.

Lipid raft assembly also plays an important role in the later steps of B cell activation, as it is required to concentrate MHC class II molecules on the B cell surface for efficient Ag presentation to T cells (24). BCR stimulation triggers lipid raft coalescence at the plasma membrane, an event that could be partly controlled by cytoskeleton remodeling, as in T cells. This was proposed, based on a quantitative proteomic analysis of lipid rafts from activated and non-activated B cells, which found several cytoskeleton-related proteins, such as actin motor proteins from the myosin family, to be accumulated in lipid rafts fractions of activated B cells. Moreover, ezrin, an adapter of the cortical actin cytoskeleton, was shown to transiently dissociate from actin and lipid rafts upon BCR stimulation, revealing its possible role in controlling lipid raft coalescence at the plasma membrane (25). Thus, activation of BCR and TCR involves early changes in the lipid environment that are linked to changes in the actin cytoskeleton, organizing signaling molecules and receptors that determine IS function.

### Endocytic trafficking

#### TCR endocyotsis and trafficking

Engagement of the TCR to MHC–peptide complexes after formation of T–APC conjugates results in its down-modulation from the cell surface, a process known to occur in an antigen dose- and time-dependent fashion. TCR down-modulation is controlled at the ISFigure 2 and thought to play a role in T cell desensitization: cSMAC formation enhances TCR signaling for low-affinity Ags, by integrating signaling molecules and co-receptors with engaged TCRs (26). Conversely, stimulation by strong agonists triggers TCR internalization and degradation from the cSMAC, which leads to the down-modulation of TCR signaling (27). TCR internalization is a clathrin-dependent process (28); however, lipid rafts also mediate internalization of TCR signaling components, such as SLP-76 (29). Down-modulation of the TCR from the cell surface can also be achieved by preventing its recycling to the plasma membrane (30) and targeting the receptor for degradation. TCR and corresponding signaling components are targeted for degradation to the lysosomal pathway by means of an ubiquitination signal, an event that was shown to occur in the IS, where ubiquitin is enriched (31). Therefore, by congregating the appropriate molecular effectors, IS formation can promote receptor internalization and degradation, thereby adapting the T cell response to the nature of the stimulus. The down-modulation of the TCR and related signaling components may further contribute to terminate IS formation and function, although the cellular aspects that drive synapse disassembly have not been addressed so far.

**Figure 2:**
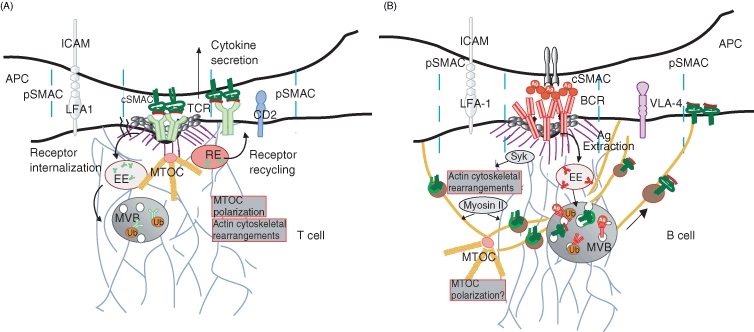
Later stages of the IS in B and T cells: vesicle trafficking and changes in polarization and membrane morphology determine effector functions Later stages of lymphocyte activation leads to a dynamic reorganization of the membrane interface characterized by the presence of two concentric regions: cSMAC, where immune-engaged receptors are found and a pSMAC containing adhesion molecules, such as LFA-1 interacting with ligands on the surface of the APC. A) TCR downstream signaling events drive the polarization of the microtubule network, highlighted by the translocation of the MTOC, toward the IS, a mechanism that serves as a guide for the targeted secretion of cytokines. At the IS, engaged TCRs are internalized from the cSMAC, where they can be recycled to the plasma membrane or targeted for degradation by ubiquitination. B) B cells engaged to membrane-bound Ag leads to its extraction, by a yet undefined mechanism. Ag–BCR complexes are transported by ubiquitin signaling into late-endocytic compartments for processing and the derived peptides are loaded onto MHC class II molecules for further presentation to CD4 T cells. Activated BCR-signaling downstream effectors such as Syk and the Myosin II motor trigger actin cytoskeleton remodeling, promoting an efficient recruitment of MHC class II/Lamp1+molecules toward the Ag−processing compartment. In both schemes, downstream effector molecules control vesicle trafficking and membrane cytoskeleton remodeling, all events dictating lymphocyte effector function.

#### BCR endocytosis and trafficking

BCR endocytosis is promoted by Ag binding. Studies with soluble Ag have shown that rapid internalization of the BCR is highly dependent on clathrin, where lipid rafts and actin cytoskeleton contribute to a lesser extent (32). However, lipid rafts could also control BCR internalization by organizing signaling cascades that modulate clathrin-mediated uptake (33). Similarly to the TCR, targeting of the BCR to late-endosomal compartments for degradation is triggered by ubiquitination of the receptor, a process occurring in the Ig*β* chain (34) and in the immunoglobulin heavy chain (35) of the BCR complex. Whether the ubiquitination of Ag-engaged BCR is also controlled at the IS, as observed in T cells, remains to be addressed. Differential BCR trafficking events, in turn, may impact IS formation by regulating the amount of receptor and associated signaling molecules at the synaptic area.

In addition to its signaling function, the BCR promotes uptake of Ag into lyso-endosomal compartments for its processing and further presentation to T cells (Figure 2) (36). The BCR-mediated uptake of membrane-tethered Ag requires the formation of an IS, allowing the gathering of Ag into the synapse, its subsequent extraction and further processing and presentation to T cells (7). Similarly to T cells, the biochemical nature of the Ag will define the downstream events of BCR endocytosis. Indeed, oligomeric Ags, such as those that are membrane-bound, were shown to trigger stronger BCR-mediated signaling and to promote more efficient endocytic trafficking of BCR–Ag complexes compared with monovalent Ag (37). Does BCR trafficking affect IS function in B cells? The cellular and molecular mechanisms that determine BCR internalization upon encounter of membrane-bound Ag have not been addressed. However, targeting of BCR–Ag complexes to late-endosomal compartments could down-regulate BCR-signaling molecules, thereby ending the phase of interaction with the APC required for Ag extraction and favoring the Ag-processing function of B cells.

#### Exocytosis at the IS

The IS can be viewed as a dynamic platform of bi-directional membrane trafficking, where endocytic and exocytic processes take place. In this sense, both helper and cytotoxic T cells were shown to secrete effector molecules such as cytokines and lytic granules, through the IS (6,38). This type of directional secretion resembles what occurs in neuronal synapses and provides a selective way to activate or destroy cells engaged in an IS. Indeed, exocytosis at the IS relies on the polarization of the microtubule organizing center (MTOC) (see cell polarity, below) and in T cells was found to occur in subdomains of the IS that were devoid of actin (39). The mechanisms underlying cytoskeleton rearrangements destined to direct membrane trafficking toward the IS are unclear; however, inactivation of ERM (ezrin–radixin–moesin) proteins, triggered upon immune receptor engagement, leads to the relaxation of the cytoskeletal cortex (25,40). Additionally, myosin IIA has been proposed to act as a link between cortical actin and microtubules, thereby facilitating granule fusion at the synapse area of NK cells (41). Altogether, these data suggest that immune receptor downstream molecules modulate the cytoskeletal cortex at synaptic interface in order to direct vesicle secretion.

Secretion at the IS of B cells engaged to membrane-bound antigen has not been reported; however, the directional secretion of proteases at the synaptic interface could be employed for Ag extraction. Alternatively, B cells might perform membrane extraction through a process known as trogocytosis, which involves intercellular exchange of membrane patches. Indeed, one could make a parallel with T helper cells, which were shown to capture MHC–peptide complexes from APCs and to present such complexes to CD4 T cells, thereby modulating the immune response (42). Both scenarios would require the targeting of effector molecules to the IS and could depend on defined membrane-trafficking events, such as lysosomal trafficking or cytoskeletal rearrangements to generate the required force for Ag extraction through trogocytosis.

Interestingly, upon activation, both B cells and T cells were shown to secrete endocytosis-derived vesicles termed exosomes (43–45), which contain MHC molecules, TCR or BCR and adhesion molecules, among others. Their secretion has been shown to stimulate immune responses *in vivo* and because of their content, exosomes are proposed to act as cellular messengers that modulate the immune response of neighboring cells (46). Whether exosomal secretion occurs at the IS and modulates this intercellular communication would be interesting to explore.

### Actin cytoskeleton dynamics at the IS

TCR engagement triggers a spreading response that involves the formation of actin-rich structures at the T cell contact site. The actin cytoskeleton serves to organize the signaling zone of the TCR (47), participating in the initial clustering of TCR–MHC–peptide complexes and integrins, therefore controlling the formation and stabilization of the IS. Live imaging of T cells revealed that actin polymerization begins at the site of TCR engagement and relies on the previous recruitment of signaling molecules such as LAT and SLP-76 (48). In general the control of actin dynamics in T cells is very complex, many of the molecules involved in this process have been described and analyzed in other reviews (49) and for simplicity are not mentioned here. The main players controlling actin polymerization at the T cell contact site require the activity of VAV proteins, particularly Vav-1, which is recruited to activated TCRs and acts as a guanine nucleotide exchange factor (GEF) for the Rho GTPases Rac and Cdc42 (Figure 1). Activation of Cdc42 was shown to promote actin polymerization at the IS, by interaction with WASP, via Arp2/3 complex (50), thus contributing to the assembly of a stable IS by organizing receptors and signaling molecules.

B cell recognition of membrane-bound Ag initiates a spreading and contraction response that directly relies on actin cytoskeleton (51), and is followed by the formation of an IS. The BCR-signaling pathways behind these actin-mediated cytoskeletal rearrangements are not fully understood, but, similarly to T cells, were shown to require activation of Rho GTPases, Rac1 (52) and Rac 2 (53), through a Vav-dependent pathway (Figure 1). In particular, Rac2 was shown to control actin-dependent membrane remodeling upon Ag engagement and is critical for B cell adhesion during interaction with membrane-bound Ags and IS formation (53).

Additionally, Rap1 GTPases, which are activated upon Ag receptor engagement of both B and T cells (54), promote lymphocyte adhesion through integrin activation and also regulate actin dynamics by recruiting Rho GTPase effector molecules (55). In B cells, Rap1 activation controls cell spreading and actin polymerization at the contact site with membrane-bound Ag, thus promoting stable synapse formation (56). Interestingly, Rap1 also binds the Par3-Par6-aPKC polarity complex in T cells and has been implicated in chemokine-induced T cell polarization (57), but its role in the IS has not been addressed. In general, the interplay between polarity cues and actin-mediated synapse assembly should provide new insights on how immune receptor signaling drives IS formation.

Actin cytoskeleton rearrangements triggered upon BCR engagement also modulate membrane-trafficking events required for efficient Ag processing and presentation in B cells. Actin-binding protein 1 (Abp1) was shown to promote receptor internalization (58) by regulating interactions with the actin cytoskeleton and endocytic machinery. We have highlighted a role for Syk and Myosin II, as key regulators of actin cytoskeleton remodeling for convergence of vesicles transporting Ag–BCR complexes and MHC II molecules into a compartment devoted to Ag processing (Figure 2) (59,60).

Altogether, actin cytoskeleton rearrangements, controlled by BCR engagement and small GTPases, promote cell adhesion and the formation of a stable IS. How these events impact the organization of receptor and signaling molecules at the lymphocyte–APC interface remains to be elucidated.

### Cell polarity: organizing vesicle trafficking

More than 20 years ago, Kupfer et al. showed that T helper cells acquire a polarized phenotype after establishing contact with B cells exposing MHC class II–peptide complexes on their cell surface (61). The polarization of T cells was characterized by the orientation of the Golgi apparatus together with the centrosome or MTOC toward the cell contact region with an APC. The main purpose of the MTOC/Golgi repositioning was to direct the secretion of Golgi-derived lymphokines from the T cell to the bound APC (38,62). Studies in cytotoxic T cells also highlighted the requirement for MTOC translocation toward the cell contact site, an event that promotes the delivery of secretory granules to the IS, thereby controlling the killer function of cytotoxic T cells (39).

The molecular events that determine lymphocyte polarity upon synapse formation remain largely unknown. The activity of Cdc42 was shown to be important for the translocation of the MTOC toward the site of interaction with APCs (63). This could rely on downstream signaling events that depend on ZAP-70, which also controls TCR-induced MTOC polarization and the subsequent recruitment of PKCθ and LAT, both required to form a functional IS (64). The inhibition of dynein–dynactin complex activity was also shown to impair MTOC polarization in T cells, resulting in a disorganized and dysfunctional IS and dramatically affecting TCR sustained signaling (65). Thus, microtubule organization is critical in determining the spatial organization of the T cell IS, probably by driving the transport of key signaling elements required for synapse formation. Additionally, actin modulators belonging to the Formin family of proteins, DIA1 and FMNL1, were shown to control MTOC polarization of T cells, maybe acting in concert with the microtubule network (66). The interplay between actin and microtubule cytoskeleton opens a new array of regulatory proteins that could modulate vesicle trafficking to regulate T cell synapse function and fate.

The IS formed between T cells and APCs also has the potential to influence later phases of T cell activation. Polarized T cells engaged to APCs lead to their asymmetric cell division, where polarity proteins are segregated differentially into the daughter cells, determining their fate as memory or effector cells (67).

Studies regarding the polarization of B cells have not been performed. Although, given the many similarities between T and B cell activation, it is possible that the partition of cellular markers after synapse formation can also determine their effector functions later on. In addition, studies showing that MHC class II–Ii complexes use microtubules to move bi-directionally suggest a possible role in Ag presentation (68). It is tempting to speculate that the polarization of the microtubule network toward the synaptic interface, where Ag is gathered, might drive vesicle trafficking toward the IS to promote efficient Ag extraction and processing.

## Conclusions

Despite the fact that T and B lymphocytes recognize different types of Ag, they undergo a similar global and dynamic reorganization of their membrane cytoskeleton interface following Ag stimulation. Their synapses show striking similarities both in terms of structure and of function, revealing conserved guidelines of polarity used to orchestrate their specific effector functions. Interestingly, similar events of membrane–cytoskeleton interactions than those observed during synapse formation have also been shown to operate during cell migration (69). These observations suggest that the use of common regulatory mechanisms may help leukocytes to coordinate their specific effector function in time and space, as highlighted in our recent report showing that the association between the MHC class II-associated invariant chain and the actin-based motor myosin II regulates both Ag processing and APC migration (70). In addition, recent evidence points to a cross talk between the BCR and toll-like receptor 9 (TLR9), showing that BCR stimulation controls the recruitment and signaling of TLR9 (71) and that Ag stimulation in the presence of TLR-ligand enhances B cell responses *in vivo*(72). The role of innate immune receptors such as TLRs in synapse formation and migration of B and T lymphocytes shall therefore now be addressed.
